# Comparison of China Reference with Different National and International References: The Prevalence of High Blood Pressure in 695,302 Children and Adolescents in a Metropolis of Yangtze River Delta, China

**DOI:** 10.1155/2021/3976609

**Published:** 2021-11-09

**Authors:** Min Zhang, Hai-Tao Zhang, Ri-Sheng Zha, Guo-Ping Gui, Di Han, Jia Hu, Hai-bing Yang, Hui Shen

**Affiliations:** ^1^Suzhou National New and Hi-Tech Industrial Development Zone Center for Disease Control and Prevention, Suzhou, Jiangsu, China; ^2^Suzhou Hospital of Integrated Traditional Chinese and Western Medicine, Suzhou, Jiangsu, China; ^3^Suzhou Center for Disease Prevention and Control, Suzhou, Jiangsu, China

## Abstract

**Objectives:**

This study aimed to compare performances of China reference and different national references on high blood pressure (HBP).

**Methods:**

A cross-sectional study including 695,302 children and adolescents aged 7 to 17 years in Suzhou, China, was conducted to determine the prevalence of HBP based on U.S., international, Europe, and China references in 2016.

**Results:**

Different percentiles of height and blood pressure were found among four references. Referring to U.S. reference, the prevalence of HBP was the highest with 26.0%, followed by International reference with 20.0%, Europe reference with 19.5%, and China reference with 19.2%. McNemar tests indicated statistically significant differences between HBP prevalence comparing China reference with the other 3 references (*P* < 0.001). The area under the curve was 0.947, 0.851, and 0.949 for U.S., international, and Europe reference, respectively. U.S. reference showed the highest sensitivity (98.2%), but the lowest specificity (91.2%), and Europe reference showed the highest kappa value (0.893).

**Conclusions:**

The prevalence of HBP varied among these four references, and the appropriate choice of reference would be important to recognize high-risk children and judge the trends of HBP prevalence in the targeted population.

## 1. Introduction

Hypertension or high blood pressure (BP), once considered a rare disease in children, is now regarded as an important public health problem worldwide [1]. High blood pressure (HBP) is associated with increased risk of target organ damage in children and adolescents [2, 3]. Importantly, numerous studies have shown that children with higher BP levels are also more likely to have persistent hypertension and increased risk for cardiovascular disease in adulthood [4, 5]; thus, early recognition of individuals with elevated BP and effective intervention in childhood will be an important strategy to reduce risk of cardiovascular diseases and mortality in adulthood.

Since the first “Report of the task force on blood pressure control in children” was published by the American Academy of Pediatrics (AAP) in 1977 [6], AAP has updated pediatric hypertension guidelines for four times until 2017 [3]. These series references have been widely applied to screen BP in children all over the world. In recent years, several country-specific or area-specific BP percentiles for children and adolescents have also been established worldwide. The European Society of Hypertension updated guidelines on high BP in children and adolescents in 2016 (hereinafter referred to as Europe reference) [7]; Europe reference referred to the reference provided by the U.S. Task Force for the 2004 “Fourth Report on the Diagnosis, Evaluation, and Treatment of High Blood Pressure in Children and Adolescents” [8]. Meanwhile, the International Child Blood Pressure References Establishment Consortium also established International BP references using nationally representative datasets from seven countries (China, India, Iran, Korea, Poland, Tunisia, and U.S.) in 2016 (hereinafter referred to as International reference) [9]. Besides, Iran, Germany, Poland, Congo, Egypt, etc. have explored country-specific or area-specific BP references [10–15]. As the first national BP reference for Chinese children considering the influence of height, the national blood pressure reference for Chinese Han children and adolescents aged 7 to 17 years was issued in 2017 [16]. It is well known that height is relevant with BP in children and adolescents [17, 18]. Several studies have applied China reference and U.S. reference to evaluate the prevalence for HBP in the same population, suggesting huge differences [19, 20]. A large number of potential hypertensive populations in China determines the importance of accurate recognition of youth with high BP [21].

The objectives of present study were to (1) compare China reference to different national and international references and (2) compare the performance of the determination for the prevalence of HBP and high normal blood pressure (HNBP) among children and adolescents aged 7 to 17 years based on data from the 2016 Health Promotion Program for Children and Adolescents (HPPCA) of Suzhou, China.

## 2. Materials and Methods

### 2.1. Study Areas and Population

All participants in the article were selected from 2016 HPPCA of Suzhou, China. This program aimed to assess health status and promote a healthy lifestyle of children and adolescents, and all potential students could take part in the HPPCA, and they could enjoy health examination and acquire health promotion and education for free. Detailed descriptions of HPPCA were ever reported previously [18, 22]. In this study, children with a previously known history of chronic illnesses such as chronic respiratory diseases, chronic renal diseases, chronic liver diseases, diabetes mellitus, and congenital heart diseases or children with significant physical deformities were not included. Meanwhile, those with new diagnostic diseases in the health examination mentioned above would also be excluded. For recruited participants, informed consent forms in writing duly signed by their guardians were collected before examination. The study was approved by the Ethics Committee of Suzhou Center for Disease Prevention and Control.

### 2.2. Measurements and Definition of HBP

Weight and height were measured with children in light clothing and without shoes. Body mass index (BMI) was calculated as weight in kilograms divided by the square of height in meters (kg/m^2^). Based on recommendation set by AAP in its recent updated guideline [3], initial systolic blood pressure (SBP) and diastolic blood pressure (DBP) were measured using validated oscillometric devices (OMRON HEM752, HBP-1300, etc.; available site: https://www.dableducational.org/index.html) after each subject had rested for at least 15 minutes in a sitting position. If the averaged oscillometric reading was at or above the 90^th^ percentile for gender, age, and height, mercury sphygmomanometer measurements were taken. BP was measured on the right arm with an appropriately sized cuff. The average value of three readings was adopted in all analyses [22].

The age- and gender-specific BMI cutoff points recommended by the Working Group on Obesity in China were utilized to define overweight and obesity [23]. BP was classified based on U.S. reference, International reference, Europe reference, and China reference [3, 7, 9, 16]. In the present study, HBP is defined as average SBP and/or DBP that is ≥95^th^ percentile for gender, age, and height of corresponding references, while HNBP is defined as average SBP or DBP levels that are ≥90^th^ percentile but <95^th^ percentile of corresponding references.

### 2.3. Statistical Methods

Participants were divided into different groups according to BMI, age, socioeconomic status (SES), gender, and height. Groups based on SES including relatively high SES and relatively low SES were distinguished based on GDP per capita of areas where individuals lived in (https://www.sztjj.gov.cn/). Groups based on height were divided into 3 groups: <25^th^, 25^th^ to 75^th^, and ≥75^th^ percentile, and in order to explore extreme height conditions, participants extremely short (<5^th^ percentile) and extremely tall (≥95^th^ percentile) were also assessed. The proportions identified as HBP based on China reference and other 3 references were compared using McNemar tests. Kappa values were calculated. The evaluation of HBP and HNBP according to China reference was considered as the “gold standard” for comparison with other 3 references. Receiver operator characteristic curve analysis was conducted to examine their discriminatory power of HBP and HNBP (yes versus no). The area under the curve (AUC), sensitivity, specificity, positive predictive value (PPV), and negative predictive value (NPV) were calculated. All statistical analyses were performed in R 3.2.2 software. The statistical significant level was defined as *P* values <0.05 (two-sided).

## 3. Results

### 3.1. Characteristics of the Sample

Total participants were 695,302, of which 53.9% were males. The percentage of subjects with HBP in each groups divided by BMI, age, SES, gender, and height is listed in Tables 1–3. A comparison of children who were at various weight statuses revealed a greater percentage of HBP in subjects who had higher BMI (*P* < 0.001). Old adolescents (13–17 years old) were more likely to have high BP than young children (7–12 years old) (*P* < 0.001; except U.S. reference; Table 1). High-SES groups have more HBP subjects than low-SES groups. Males had more HBP than females (*P* < 0.001), and more females were HBP when China reference were selected (Table 2). Shorter children had HBP more than taller ones according to China reference. An opposite effect was observed for the other 3 references. This situation was more obvious in extremely short and tall children and adolescents (Table 3). Tables S1–S3 show the percentage of subjects with HBP and HNBP in each groups divided by BMI, age, SES, gender, and height.

### 3.2. Characteristics of the Sample

**Figure 1:**
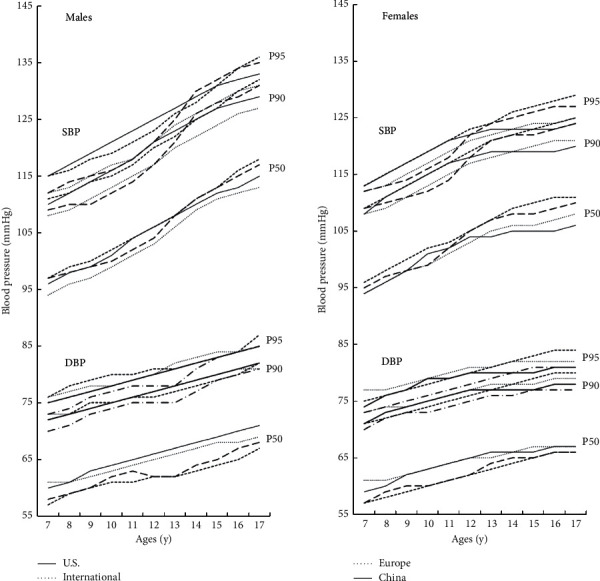
The 50^th^, 90^th^, and 95^th^ percentiles of 4 BP references for males and females aged 7 to 17 years at median height.

Figure 1 shows the 50^th^, 90^th^, and 95^th^ percentiles of 4 BP references for males and females aged 7 to 17 years at median height. Generally, the pattern of differences was different in males and females. For example, for males, the China reference-95^th^ SBP was the highest before 14 years of age and was surpassed followed by U.S. (at 14 years of age) and Europe (at 16 years of age) references. For females, the China reference-95^th^ SBP was on par with Europe reference, higher than the other 2 references before 12 years of age, and it was surpassed followed by Europe (at 12 years of age), U.S. (at 13 years of age), and International (at 15 years of age) references.  Figure S1 shows different height values of the four references. The height values reported in China reference were similar to those reported in the other 3 references in younger children and lower in older adolescents, and differences were most significant in those 17 years old.

### 3.3. Prevalence of HBP Based on Four References

In Figure S2, BP in children was evaluated according to U.S. (2017), International (2016), Europe (2016), and China (2017) references. Major differences were observed in the classification of HBP. Referring to U.S. reference, the prevalence of HBP was the highest with 26.0%, followed by International reference (20.0%), Europe reference (19.5%), and China reference (19.2%). In correspondence to the increase in the prevalence of HBP, the prevalence of normal blood pressure (NBP) decreased. The percentage of NBP was the highest with 68.5% referring to China reference and the lowest with 60.8% referring to U.S. reference.

### 3.4. Consistency Analysis for Identifying HBP Based on Four References

The proportions of children categorized by every method were calculated, and McNemar tests indicated statistically significant differences between the HBP prevalence in comparing China reference with other 3 references (*P* < 0.001, Table 4). Compared with China reference (“gold standard”), the U.S. reference showed AUC 0.947, sensitivity 98.2%, specificity 91.2%, PPV 72.6%, NPV 99.5%, and kappa value 0.788, whereas the International reference showed AUC 0.851, sensitivity 76.7%, specificity 93.5%, PPV 73.6%, NPV 94.4%, and kappa value 0.691. The performance of Europe reference was excellent, with AUC 0.949, sensitivity 92.1%, specificity 97.8%, PPV 90.7%, NPV 98.1%, and kappa value 0.893 (Table 5). Consistency results of HBP and HNBP using these 4 references are shown in Tables S4 and S5.

## 4. Discussion

Estimation of BP in children and adolescents should be a chief vital domain of global healthcare. The BP reference for children and adolescents was firstly developed on the basis of American children. The First Report stated the BP normal range (from 5^th^ to 95^th^ percentile) for boys and girls aged over 2 years, and including data were collected almost exclusively from white children [6]. The Second Report broadened the ages' ranges from birth to 18 years, and included data in more representative children (white, black, and Mexican-American) [24]. The Third Report was the first to include body height percentile [25]. The Fourth Report updated BP reference including new data from the 1999 to 2000 U.S. National Health and Nutrition survey [8]. The Fifth Report established new pediatric BP tables based on normal-weight children and presented a simplified screening table for identifying abnormal BP needing further evaluation [3]. These series references issued by AAP have been extensively used all over the world; however, children in the United States represented a more heterogeneous group and differed from populations in other continents. Thus, other areas began to establish country-specific or area-specific references inspired by AAP.

Similarly, China also experienced different stages of BP reference establishment, which had made rapid development, especially in the past decade. In 2007, Ji et al. established age- and gender-specific BP cutoffs in Chinese children and adolescents 6–22 years old based on data of the 2005 Chinese National Survey on Students' Constitution and Health (CNSSCH). In 2010, on the basis of data from eleven large-scale cross-sectional BP surveys including four municipalities and seven provinces including 112,227 subjects aged 3 to 18 years, a new BP reference was developed and presented in an age- and gender-specific BP cutoffs [26]. In 2013, a reference sample of 11,952 subjects aged 7–17 years from the Chinese Health and National Survey 1999–2009 after excluding overweight and obese children was applied to provide a reference BP table for age, gender, and height in Chinese children, which was the first height-specific BP reference to propose for Chinese children and adolescents, and it was also the first reference based on data excluding overweight and obese children in China [27]. In 2017, Mi et al. updated the 2010 BP reference, and the normal range for BP (50^th^, 90^th^, 95^th^, and 95^th^ percentile) in various body height percentiles using the same data in 2010 BP references was presented [28]. Meanwhile, Ma et al. published national blood pressure reference based on data obtained on 197,430 children aged 7 to 17 years who participated in the 2010 CNSSCH, which was the first national BP reference issued by the National Health Commission of the People's Republic of China and named as China reference in the present study for Chinese children based on age, sex, and height by using large-scale and nationally representative data [16]. To our knowledge, this is the first study to compare China reference with other country-specific or area-specific references. We observed different patterns in these references. In this rapidly developed Chinese setting, the prevalence of HBP was the highest referring to U.S. reference with 26.0%, followed by International reference (20.0%), Europe reference (19.5%), and China reference (19.2%). Indeed, the positive relation between BP and height means BP reference in children without consideration of proper height is inaccurate [22, 29], particularly for children who are very short (5^th^ percentile) or very tall (95^th^ percentile) (Table 3). Previous studies indicated that the influence of height on BP may even be greater than age [30]. In this study, we found that there were great differences in height for both males and females among China and other references (Figure S1), and the gap increased in late puberty, especially. Meanwhile, the BP values also showed differences among these four references (Figure 1). Different growth curves for children and adolescents from different population would affect the establishment of appropriate boundaries for HNBP and HBP, which would further reduce the accuracy of BP evaluation. Ethnic, racial, and geographic differences might also explain the variability of height and BP in the populations studied in China, United States, and other areas. These differences may be caused by multiplex determinants including genetic and environmental factors and gene-environment interactions. Weight or BMI is a key determinant of BP in children (Table 1), and children with higher BMI or weight are more likely to be hypertensive individuals [19, 31, 32]. In this study, two references based on nonoverweight/obesity populations (U.S. and International references) resulted in higher portions of HBP, whereas the other two references (Europe and China references) showed lower prevalence of HBP. A study used the Fourth Report nonoverweight/obesity reference and Fourth Report reference to evaluate the same targeted children, and higher prevalence of elevated BP was found when the Fourth Report nonoverweight/obesity reference was used [33]. Considering the subhealthy situation of overweight and obesity, references based on nonoverweight/obesity populations may be more appropriate and representative to evaluate different categories of BP in children. Although China reference establishment did not remove overweight/obesity children, direct use of U.S. or International references would bring in some misclassifications as a result of different definitions of overweight and obesity [34].

Numerous studies have shown that children with higher BP levels have increased risk for cardiovascular disease in adulthood [4]. Although a number of functional, imaging, and autopsy studies have demonstrated BP-related cardiovascular damage in children and adolescents [35, 36], the exact effect level and duration of elevated BP that cause target organ damage have not been understood. At present, a statistical definition is still the principal method for childhood high BP evaluation. High BP in children and adolescents is defined at or above the 95^th^ percentile of the distribution, and high normal BP is defined by the upper 10% of the distribution. In the future, long-term studies should be conducted to explore clinical markers for early cardiovascular disease that include increased carotid intima-media thickness, left ventricular mass and pulse wave velocity, flow-mediated dilatation, retinal vascular narrowing, or increased cardiovascular biomarkers (interleukin-6, fibrinogen, and so on) [37–39], which would be useful for the establishment of HBP definition in children and adolescents.

Early interventions would be effective in controlling hypertension development and achieving an ideal BP level. Lifestyle and nonpharmacologic interventions are recommended as the primary method to lower and control BP. The dietary approaches to stop hypertension including a diet that is high in fruits, vegetables, low-fat milk products, whole grains, fish, poultry, nuts, and lean red meats are suggested; they also include a limited intake of sugar and sweets along with lower sodium intake. Besides, observational data support the benefits of physical activity on BP. Programs that combine diet and physical activity can have a considerable effect on BP levels, as is shown in several studies designed to prevent childhood obesity which is an important risk factor for elevated BP.  Meanwhile, proper sleep and stress reduction are also recommended. Although antihypertensive drug therapy is a further possibility if insufficient response to lifestyle modifications exists, drug is still recommended to use cautiously. Long-term studies on the safety of antihypertensive medications in children and their impact on future cardiovascular disease are limited. Randomized clinical trial and long-term follow-up studies are essential to acquire more knowledge about benefits and undesirable effects of antihypertensive drug in children and adolescents. Our study has several strengths. This study is the first to compare new China reference with other country-specific or area-specific references. The highest amount of potential hypertensive populations in China determines the importance of accurate recognition of youth with high BP. The large sample size is an additional study strength together with the well-standardized study anthropometric and BP assessments made by qualified staff.

Limitations of the present study should be noted. First, although three BP readings were recorded, it was only in a single visit. BP decreases with repeated measurements over several visits. This systematic overestimation might influence the evaluation of HBP and HNBP accurately. Besides, since participants were from only one region of Eastern China, the generalizability of the present findings might be limited to represent corresponding regions in China to some extent, and the results of the present study need to be confirmed in lager areas.

## Figures and Tables

**Table 1:** Prevalence of high blood pressure (%) in the study population based on the 4 reference systems stratified by weight status or age.

	Weight status (BMI)				Age (years)		
	Normal	Overweight	Obese	*P* value	7–12	13–17	*P* value
U.S.	22.1	31.8	41.8	<0.001	26.0	26.0	0.997
International	16.3	25.9	34.6	<0.001	18.5	25.6	<0.001
Europe	16.4	24.1	32.5	<0.001	18.7	22.4	<0.001
China	16.6	23.0	29.8	<0.001	17.8	24.3	<0.001

*P* value represents differences among categories.

**Table 2:** Prevalence of high blood pressure (%) in the study population based on the 4 reference systems stratified by socioeconomic status or gender.

	Socioeconomic status			Gender		
	Low	High	*P* value	Males	Females	*P* value
U.S.	24.5	27.7	<0.001	27.1	24.7	<0.001
International	19.6	20.4	<0.001	21.8	17.9	<0.001
Europe	18.5	20.6	<0.001	20.1	18.8	<0.001
China	18.2	20.3	<0.001	18.8	19.7	<0.001

*P* value represents differences among categories.

**Table 3:** Prevalence of high blood pressure (%) in the study population based on the 4 reference systems stratified by height.

	<25^th^ percentile	25^th^ to <75^th^ percentile	≥75^th^ percentile	*P* value	<5^th^ percentile	≥95^th^ percentile
U.S.	24.0	25.7	26.9	<0.001	20.7	29.3
International	17.7	19.3	21.4	<0.001	14.5	23.2
Europe	19.1	19.4	19.8	<0.001	16.1	22.0
China	20.6	19.9	18.0	<0.001	21.0	16.8

**Table 4:** Consistency analysis for identifying high blood pressure based on China and the other 3 reference systems.

		China								
		Males			Females			Total		
		+	−	*P* value	+	−	*P* value	+	−	*P* value
U.S.	+	69,439 (98.8%)	32,179 (10.6%)		61,778 (97.6%)	17,415 (6.8%)		131,217 (98.2%)	49,594 (8.8%)	
	−	857 (1.2%)	272,156 (89.4%)	<0.001	1493 (2.4%)	239,985 (93.2%)	<0.001	2350 (1.8%)	512,141 (91.2%)	<0.001

International	+	55,312 (78.7%)	26,329 (8.7%)		47,080 (74.4%)	10,308 (4.0%)		102,392 (76.7%)	36,637 (6.5%)	
	−	14,984 (21.3%)	278,006 (91.3%)	<0.001	16,191 (25.6%)	247,092 (96.0%)	<0.001	31,175 (23.3%)	525,098 (93.5%)	<0.001

Europe	+	66,019 (93.9%)	9199 (3.0%)		56,977 (90.1%)	3434 (1.3%)		122,996 (92.1%)	12,633 (2.2%)	
	−	4277 (6.1%)	295,136 (97.0%)	<0.001	6294 (9.9%)	253,966 (98.7%)	<0.001	10,571 (7.9%)	549,102 (97.8%)	<0.001

**Table 5:** Performance of the 3 other methods for the identification of high blood pressure.

	AUC (95% CI)	Sensitivity	Specificity	PPV	NPV	Kappa value
*Males*						
U.S.	0.941 (0.940, 0.942)	0.988	0.894	0.683	0.997	0.753
International	0.850 (0.848, 0.852)	0.787	0.913	0.678	0.949	0.659
Europe	0.954 (0.953, 0.956)	0.939	0.970	0.878	0.986	0.885

*Females*						
U.S.	0.954 (0.953, 0.955)	0.976	0.932	0.780	0.994	0.830
International	0.852 (0.850, 0.854)	0.744	0.960	0.820	0.939	0.730
Europe	0.944 (0.942, 0.945)	0.901	0.987	0.943	0.976	0.903

*Total*						
U.S.	0.947 (0.946, 0.948)	0.982	0.912	0.726	0.995	0.788
International	0.851 (0.849, 0.852)	0.767	0.935	0.736	0.944	0.691
Europe	0.949 (0.948, 0.950)	0.921	0.978	0.907	0.981	0.893

## Data Availability

The data used to support the findings of this study are available from the corresponding author upon request.

## Abbreviations

abrBP: Blood pressure
HBP: High blood pressure
AAP: American Academy of Pediatrics
U.S.: The United States
HNBP: High normal blood pressure
HPPCA: Health Promotion Program for Children and Adolescents
BMI: Body mass index
SBP: Systolic blood pressure
DBP: Diastolic blood pressure
SES: Socioeconomic status
AUC: Area under the curve
PPV: Positive predictive value
NPV: Negative predictive value
NBP: Normal blood pressure
CNSSCH: Chinese National Survey on Students' Constitution and Health.

## Supplementary Materials

Supplementary Materials Supplementary Table 1. Prevalence of high blood pressure and high normal blood pressure (%) in the study population based on the 4 reference systems stratified by weight status or age. Supplementary Table 2. Prevalence of high blood pressure and high normal blood pressure (%) in the study population based on the 4 reference systems stratified by socioeconomic status or gender. Supplementary Table 3. Prevalence of high blood pressure and high normal blood pressure (%) in the study population based on the 4 reference systems stratified by height. Supplementary Table 4. Consistency analysis for identifying high blood pressure and high normal blood pressure based on China and the other 3 reference systems. Supplementary Table 5. Performance of the 3 other methods for the identification of high blood pressure and high normal blood pressure. Supplementary Figure 1. The 10^th^, 50^th^, 90^th^, and 95^th^ percentiles of height for males and females aged 7 to 17 years based on the 4 BP references. Supplementary Figure 2. Prevalence of normal BP (NBP), high-normal BP (HNBP), and high BP (HBP) in the studied children and adolescents based on the 4 references.
