# Supplementary material for: Comparison of China Reference with Different National and International References: The Prevalence of High Blood Pressure in 695,302 Children and Adolescents in a Metropolis of Yangtze River Delta, China
Source: Int J Hypertens. 2021 Nov 9;2021:3976609. doi: 10.1155/2021/3976609 (PMC8595015; doi:10.1155/2021/3976609)
Supplement: Supplementary Materials — Supplementary Table 1. Prevalence of high blood pressure and high normal blood pressure (%) in the study population based on the 4 reference systems stratified by weight status or age. Supplementary Table 2. Prevalence of high blood pressure and high normal blood pressure (%) in the study population based on the 4 reference systems stratified by socioeconomic status or gender. Supplementary Table 3. Prevalence of high blood pressure and high normal blood pressure (%) in the study population based on the 4 reference systems stratified by height. Supplementary Table 4. Consistency analysis for identifying high blood pressure and high normal blood pressure based on China and the other 3 reference systems. Supplementary Table 5. Performance of the 3 other methods for the identification of high blood pressure and high normal blood pressure. Supplementary Figure 1. The 10th, 50th, 90th, and 95th percentiles of height for males and females aged 7 to 17 years based on the 4 BP references. Supplementary Figure 2. Prevalence of normal BP (NBP), high-normal BP (HNBP), and high BP (HBP) in the studied children and adolescents based on the 4 references. [file 3976609.f1.docx]

**Supplementary Tables**

**Supplementary Table 1**. Prevalence of high blood pressure and high normal blood pressure (%) in the study population based on 4 reference systems stratified by weight status or age.

|  |  | Weight status (BMI) | | | |  | Age(years) | | |
| --- | --- | --- | --- | --- | --- | --- | --- | --- | --- |
|  |  | Normal | Overweight | Obese | *P*-value |  | 7-12 | 13-17 | *P*-value |
| U.S. |  | 34.7 | 46.6 | 56.6 | <0.001 |  | 38.5 | 41.5 | <0.001 |
| International |  | 32.1 | 44.6 | 54.5 | <0.001 |  | 34.9 | 43.9 | <0.001 |
| Europe |  | 28.3 | 38.7 | 48.2 | <0.001 |  | 31.0 | 37.3 | <0.001 |
| China |  | 28.0 | 37.1 | 45.5 | <0.001 |  | 29.6 | 38.8 | <0.001 |

*P*-value for differences among categories.

**Supplementary Table 2**. Prevalence of high blood pressure and high normal blood pressure (%) in the study population based on 4 reference systems stratified by socioeconomic status or gender.

|  |  | Socioeconomic status | | |  | Gender | | |
| --- | --- | --- | --- | --- | --- | --- | --- | --- |
|  |  | Low | High | *P*-value |  | Males | Females | *P*-value |
| U.S. |  | 37.5 | 41.0 | <0.001 |  | 41.0 | 37.1 | <0.001 |
| International |  | 35.5 | 38.2 | <0.001 |  | 38.7 | 34.5 | <0.001 |
| Europe |  | 30.8 | 34.0 | <0.001 |  | 33.2 | 31.2 | <0.001 |
| China |  | 30.1 | 33.1 | <0.001 |  | 31.1 | 32.0 | <0.001 |

*P*-value for differences among categories.

**Supplementary Table 3**. Prevalence of high blood pressure and high normal blood pressure (%) in the study population based on 4 reference systems stratified by Height.

|  |  | <25^th^ percentile | 25^th^ -<75^th^ percentile | ≥75^th^ percentile | *P*-value | <5^th^ percentile | ≥95^th^ percentile |
| --- | --- | --- | --- | --- | --- | --- | --- |
| U.S. |  | 37.1 | 38.7 | 40.3 | <0.001 | 33.0 | 43.5 |
| International |  | 33.1 | 35.7 | 39.0 | <0.001 | 29.3 | 41.7 |
| Europe |  | 31.3 | 31.9 | 33.1 | <0.001 | 27.9 | 35.7 |
| China |  | 32.7 | 31.9 | 30.8 | <0.001 | 33.2 | 30.3 |

**Supplementary Table 4**. Consistency analysis for identifying high blood pressure and high normal blood pressure based on China and other 3 reference systems.

|  |  | China |  |  |  |  |  |  |  |  |  |  |
| --- | --- | --- | --- | --- | --- | --- | --- | --- | --- | --- | --- | --- |
|  |  | Males |  |  |  | Females |  |  |  | Total |  |  |
|  |  | + | - | *P*-value |  | + | - | *P*-value |  | + | - | *P*-value |
| U.S. | + | 115324(88.5%) | 29678(11.5%) |  |  | 100231(97.6%) | 18644(8.6%) |  |  | 2115580(98.3%) | 56735(11.9%) |  |
|  | - | 1295(1.1%) | 115324(98.9%) | <0.001 |  | 2495(2.4%) | 199301(91.4%) | <0.001 |  | 3765(1.7%) | 419222 (88.1%) | <0.001 |
| International | + | 115324(98.9%) | 29678(11.5%) |  |  | 99339(96.7%) | 11278(5.2%) |  |  | 214663(97.9%) | 40956(8.6%) |  |
|  | - | 1295(1.1%) | 228334(88.5%) | <0.001 |  | 3387(3.3%) | 206667(94.8%) | <0.001 |  | 4682(2.1%) | 435001(91.4%) | <0.001 |
| Europe | + | 113798(97.6%) | 10640(4.1%) |  |  | 94463(92.0%) | 5738(2.6%) |  |  | 208261(94.9%) | 16378(3.4%) |  |
|  | - | 2821(2.4%) | 247372(95.9%) | <0.001 |  | 8263(8.0%) | 212207(97.4%) | <0.001 |  | 11084(5.1%) | 459579(96.6%) | <0.001 |

**Supplementary Table 5**. Performance of the 3 other methods for the identification of high blood pressure and high normal blood pressure.

|  | AUC(95%CI) | Sensitivity | Specificity | PPV | NPV | Kappa-value |
| --- | --- | --- | --- | --- | --- | --- |
| Males |  |  |  |  |  |  |
| U.S. | 0.921(0.920, 0.922) | 0.989 | 0.795 | 0.795 | 0.989 | 0.819 |
| International | 0.937(0.936, 0.938) | 0.989 | 0.885 | 0.795 | 0.994 | 0.819 |
| Europe | 0.967(0.967, 0.968) | 0.976 | 0.959 | 0.914 | 0.989 | 0.918 |
| Females |  |  |  |  |  |  |
| U.S. | 0.945(0.944, 0.946) | 0.976 | 0.914 | 0.843 | 0.988 | 0.855 |
| International | 0.958(0.957, 0.958) | 0.967 | 0.948 | 0.898 | 0.984 | 0.897 |
| Europe | 0.947(0.946, 0.948) | 0.920 | 0.974 | 0.943 | 0.963 | 0.899 |
| Total |  |  |  |  |  |  |
| U.S. | 0.932(0.931, 0.932) | 0.998 | 0.881 | 0.974 | 0.991 | 0.811 |
| International | 0.946(0.946, 0.947) | 0.979 | 0.914 | 0.840 | 0.989 | 0.855 |
| Europe | 0.958(0.957, 0.958) | 0.949 | 0.966 | 0.927 | 0.976 | 0.909 |

**Supplementary Figure legends**

**Supplementary Figure 1.** The 10^th^, 50^th^, 90^th^, and 95^th^ percentiles of height for males and females aged 7 to 17 years based on 4 BP references.

**Supplementary Figure 2.** Prevalence of normal BP (NBP), high normal BP (HNBP), and high BP (HBP) in studied children and adolescents based on 4 references.


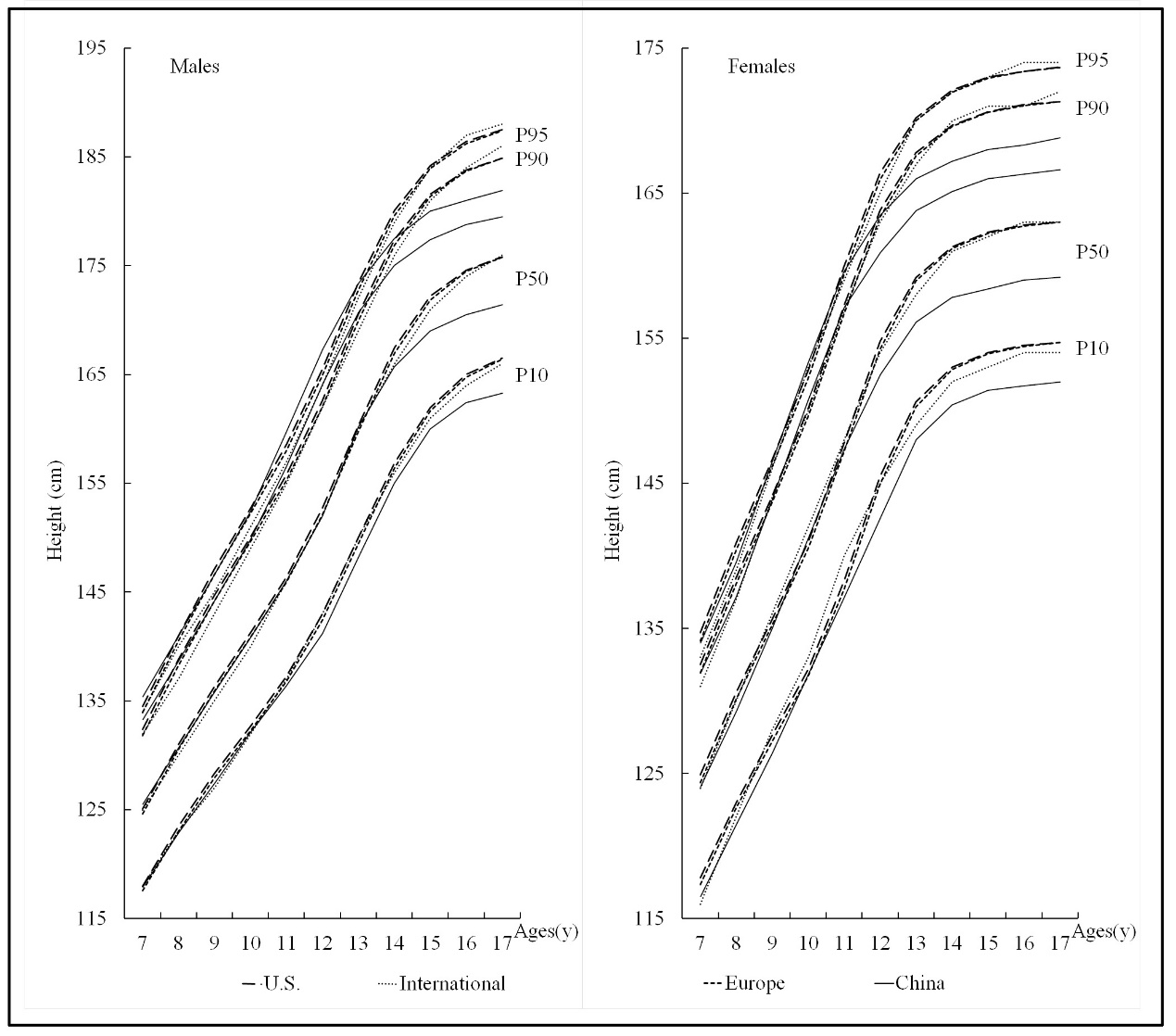


**Supplementary Figure 1.**


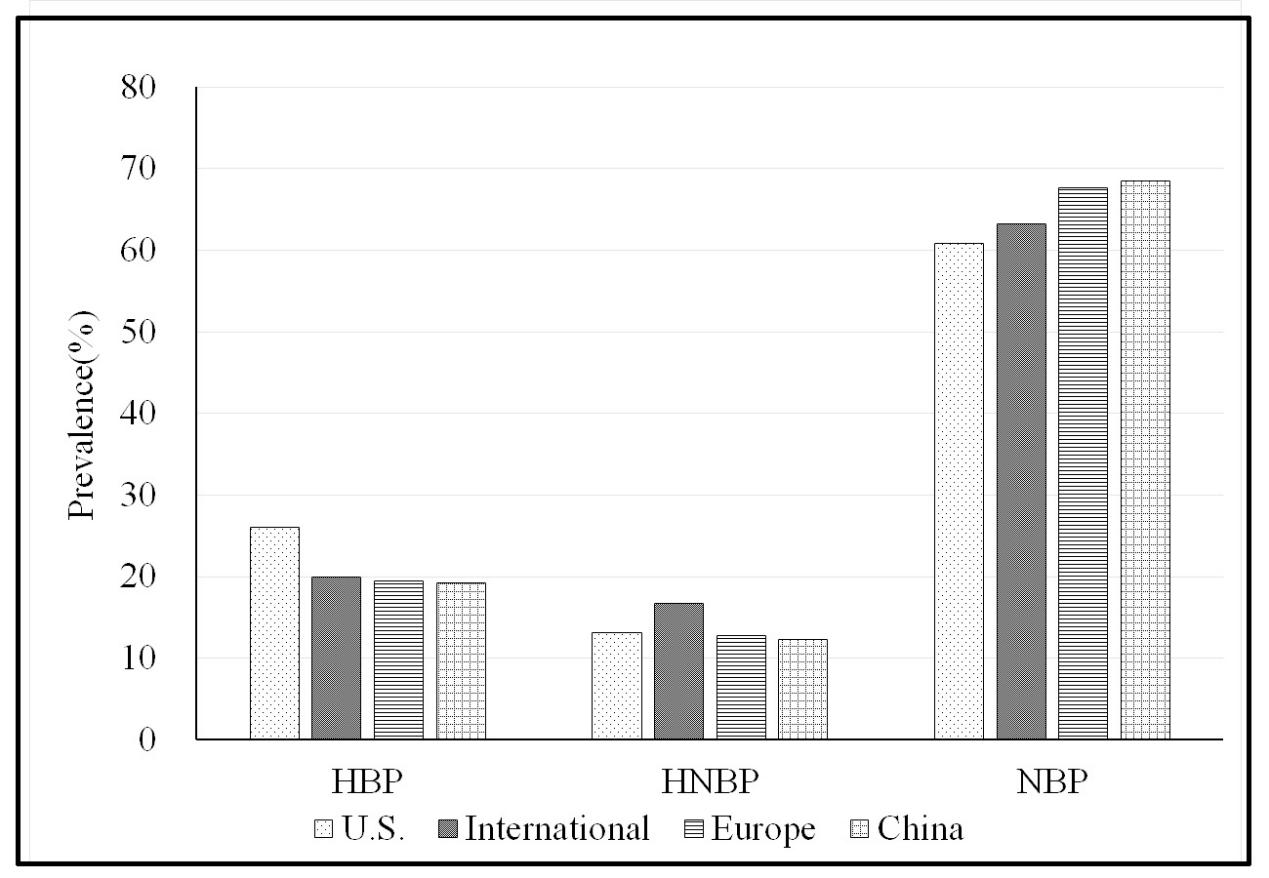
**Supplementary Figure 2.**
